# Intimate intertwining of the pathogenesis of hypoxia and systemic sclerosis: A transcriptome integration analysis

**DOI:** 10.3389/fimmu.2022.929289

**Published:** 2022-10-31

**Authors:** Xinglan He, Yaqian Shi, Zhuotong Zeng, Bingsi Tang, Xuan Xiao, Jiangfan Yu, Puyu Zou, Jiani Liu, Yangfan Xiao, Yangyang Luo, Rong Xiao

**Affiliations:** ^1^ Hunan Key Laboratory of Medical Epigenetics, Department of Dermatology, The Second Xiangya Hospital, Central South University, Changsha, China; ^2^ Department of Anesthesiology, Clinical Nursing Teaching and Research Section, The Second Xiangya Hospital, Central South University, Changsha, China; ^3^ Department of Dermatology, Hunan Children’s Hospital, Changsha, China

**Keywords:** hypoxia, systemic sclerosis, oxidative stress, PPI, crosstalk

## Abstract

**Objectives:**

Systemic sclerosis (SSc) is an autoimmune disease caused by various pathogenic factors, including hypoxia. Hypoxia stimulates the production of the extracellular matrix to promote fibrosis. However, the integrated function and the underlying mechanism of hypoxia in SSc are unclear.

**Methods:**

In the present study, we used Agilent SurePrint G3 Human Gene Expression v3 for the transcriptional sequencing of fibroblasts with and without hypoxia to detect differentially expressed genes (DEGs) in hypoxia. We analyzed the results with the transcriptome data of SSc lesions (GSE95065) to select the co-DEGs. Then, Gene Ontology and Kyoto Encyclopedia of Genes and Genomes enrichment analyses were performed on the basis of the co-DEGs using the R package ClusterProfiler, which showed that hypoxia and cross talk of hypoxia with other pathogenic factors are involved in the pathogenesis of SSc. Furthermore, we constructed a protein–protein interaction (PPI) network of co-DEGs and screened two significant functional expression modules.

**Results:**

We identified nine hub genes (ALDH1A1, EGF, NOX4, LYN, DNTT, PTGS2, TKT, ACAA2, and ALDH3A1). These genes affect the pentose phosphate pathway, oxidative stress, and lipolysis.

**Conclusion:**

Our study provides insights into the mechanisms underlying the effects of hypoxia on SSc pathogenesis, which will help to better understand SSc pathogenesis and develop new therapeutic strategies for SSc.

## Introduction

Systemic sclerosis (SSc) is an autoimmune disease that exhibits sexual dimorphism, and women have a higher incidence of SSc than men. SSc is associated with a high mortality rate and poor quality of life due to lung and heart involvement (1). The clinical presentation of SSc is characterized by vascular lesions, immune disorders, and anomalous fibrosis of the skin and other organs. The mechanisms underlying the SSc pathogenesis are not clear (2). However, it is likely that vascular lesions trigger the onset of SSc because the Raynaud phenomenon, which involves structural changes to the microvasculature, often appears as the initial manifestation of the disease (3). These vascular lesions may, in turn, lead to hypoxia.

As previously mentioned, hypoxia is considered to be involved in SSc pathogenesis. Reduced vessel density and loss of capillaries lead to impaired tissue oxygenation. Hypoxia triggers fibrosis, and chronic hypoxia often occurs in fibrotic diseases. In addition, the reduced oxygen supply stimulates the excessive deposition of the extracellular matrix and production of vascular endothelial growth factor, which promotes fibrosis by interacting with platelet-derived growth factor receptors directly. The excessive deposition of extracellular matrix aggravates angiopathy and hypoxia, which further accelerates fibrosis, similar to the pathogenesis of SSc (4, 5).

The mechanism underlying hypoxia in SSc is still unclear. Several studies have reported that hypoxia can cause fibrosis in SSc due to the production of hypoxia-inducible factors, which detect and respond to hypoxia (6–9). However, studies have not clarified the comprehensive effects of hypoxia on SSc pathogenesis and the mechanisms thereof.

**Figure 1:**
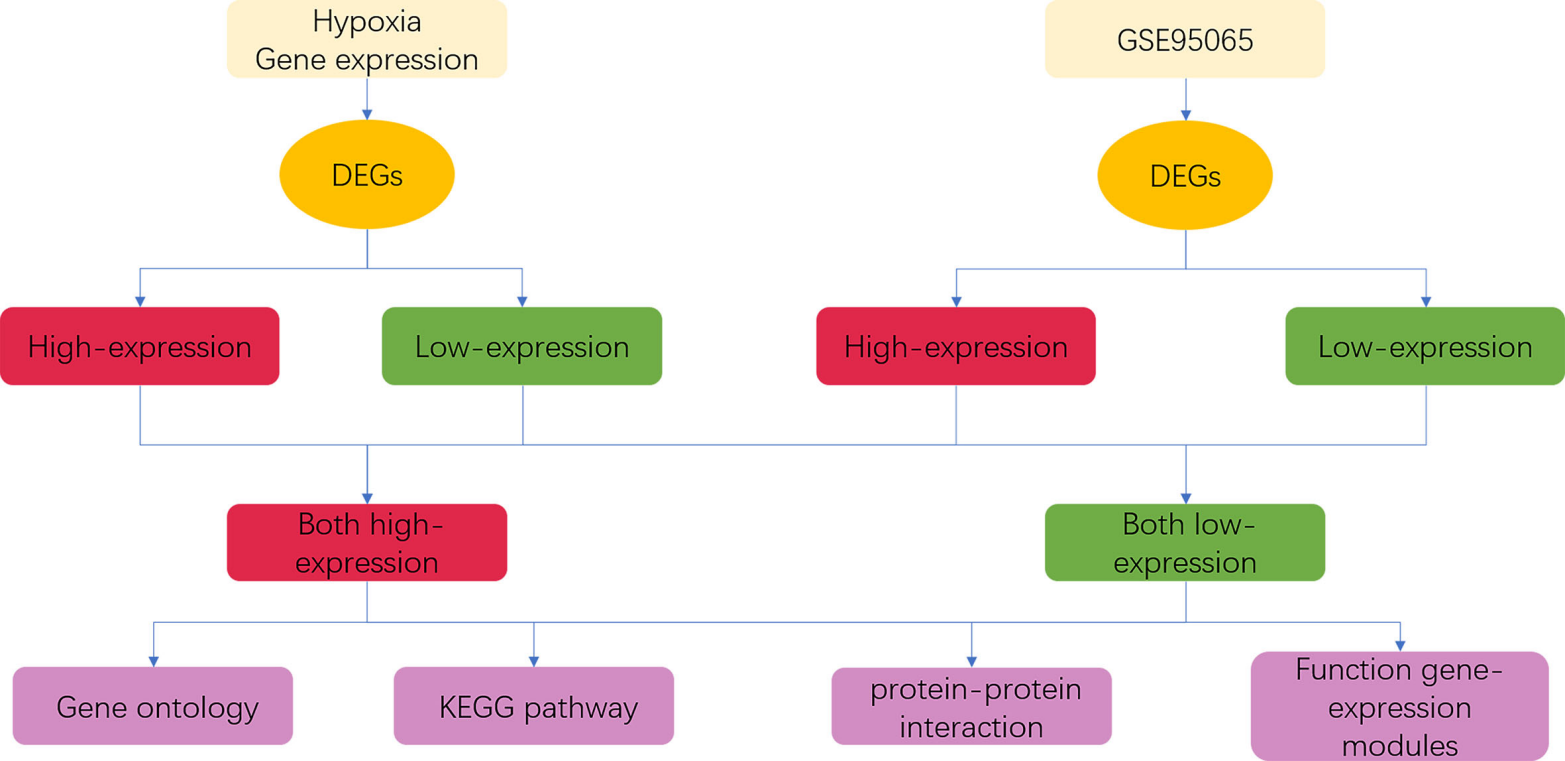
Procedure of bioinformatics analysis. DEGs, differentially expressed genes.

In the present study, we analyzed two differential transcriptomic data: expression data of fibroblasts with and without hypoxia, and expression data from skin biopsies of patients with SSc from GSE95065. We used Agilent SurePrint G3 Human Gene Expression v3 for the transcriptional sequencing of fibroblasts with and without hypoxia. The transcriptional data from SSc lesions were derived from the GSE95065 dataset (15 skin lesions from patients with SSc and 18 skin samples from controls) in the Gene Expression Omnibus (GEO) database. Then, we performed Gene Ontology (GO) and Kyoto Encyclopedia of Genes and Genomes (KEGG) pathway analyses, protein–protein interaction (PPI), hub–gene inference, and functional transcriptional module analysis of differentially expressed genes (DEGs) to explore the role of hypoxia in SSc pathogenesis. The flowchart of bioinformatics analysis is shown in **Figure 1**.

## Methods

### Study subjects

Control tissue explants are from the dermatological outpatient operating room. After three times of iodine disinfection and one time of alcohol disinfection, Full-thickness skin about 1 × 0.5 cm in size from forearm was cut with the aseptic operation to an Eppendorf (EP) tube, containing 1% double anti-sterile phosphate buffered solution (PBS), and brought into the laboratory with an ice box. Then, the explants were incubated at 37°C and 5% CO_2_ in dulbecco's modified eagle medium (DMEM) (Gibco, Carlsbad, CA, USA) supplemented with 10% Fetal Bovine Serum (FBS) (Biological Industries, Kirbuta Beit Haemek, Israel) and 1% penicillin-streptomycin (Gibco) to culture skin fibroblasts. In the hypoxia group, when the cells covered almost 60% of the 25-mm^2^ culture bottle, the tri-gas incubator was used to simulate hypoxia (37°C, 5% CO_2_, and 1% O_2_) for 24 h. The control group was cultured in the incubator under similar conditions (37°C and 5% CO_2_). The information of the control tissue explants is shown in **Table 1**.

**Table 1:** Information of control tissue explants.

Number	Gender	Age	Biopsy Site
1	Male	36	Forearm
2	Female	47	Forearm
3	Female	61	Forearm

### Microarray and data analysis

Total RNA was quantified using NanoDrop ND-2000 (Thermo Fisher Scientific, Waltham, MA, USA), and RNA integrity was assessed using Agilent Bioanalyzer 2100 (Agilent Technologies, Palo Alto, CA, USA). Sample labeling, microarray hybridization, and washing were performed according to the manufacturer’s instructions. Briefly, total RNA was transcribed to double-strand complementary DNA (cDNA), which was synthesized into cRNA and labeled with cyanine-3-CTP. The labeled cRNAs were hybridized onto the microarray. After washing, the arrays were scanned using the Agilent Scanner G2505C (Agilent Technologies).

The Feature Extraction software (version 10.7.1.1; Agilent Technologies) was used to analyze array images and to obtain raw data. GeneSpring (version 13.1; Agilent Technologies) was used to analyze the raw data. The raw data were normalized using the quantile algorithm. Probes that had 100% of the values flagged as “detected” under at least one condition were selected for further analysis. DEGs were identified through fold change and p-value calculated using the paired *t*-test in the limma package. The threshold values of upregulated and downregulated genes were set as |log FC| ≥ 0.585 and p ≤ 0.05.

### Acquisition and processing of expression spectrum data

The original gene expression dataset GSE95065 was downloaded from the GEO public database. R software (version 4.0.0; R Foundation for statistical computing, Vienna, Austria) was used to analyze and pre-process the expression matrix with the robust multiarray average method (8). Pre-processed data of the DEGs were selected using the limma package. The threshold values of upregulated and downregulated genes were set as |log FC| ≥ 0.585 and p ≤ 0.05.

### GO and KEGG enrichment

GO and KEGG enrichment analyses were performed for co-upregulated and co-downregulated genes using the R package clusterProfiler (10). GO analysis was performed using EnrichGO function in the R package “clusterProfiler”. KEGG analysis was performed using the EnrichKEGG function of the R package “clusterProfiler”. P < 0.05 was considered statistically significant. The results of the enrichment analysis were visualized using the R package ggplot2.

### Construction of PPI network and related analysis

PPI analysis was performed to explain the interactions among DEGs in SSc pathogenesis. We integrated co-upregulated and co-downregulated genes to construct PPI networks using STRING 11.0 (https://string-db.org/) (11). The results were imported to Cytoscape 3.6.0. cytoHubba app (http://apps.cytoscape.org/apps/cytohubba) was used to select the hub genes (12) by five topological analysis methods, including maximal clique centrality (MCC), maximum neighborhood component (MNC), degree, edge percolated component (EPC), and radiality. The first two modules were applied in Cytoscape using the MCODE plugin (13).

### Quantitative real-time RT-PCR

Quantitative real-time RT-PCR was carried out as previously described with minor modifications (14). Briefly, total RNA of 1 µg was reversely transcribed in a 20 μl of reaction using Evo M-MLV RT Premix for qPCR (AG, Changsha, China, code no: AG11706) according to the manufacturer’s protocol. The reaction products were then diluted with 40 μl of distilled water. The real-time PCR reaction was used of 2 μl of diluted reverse transcription product, 10 µl of 2× SYBR^®^ Green Pro Taq HS Premix (SYBR^®^ Green Premix Pro Taq HS qPCR Kit, code no: AG11606), and 0.6 µl of forward and reverse primers (0.3 μM). The reaction was performed in a Light Cycler@ 480 II Sequence Detection System (Roche, Basel, Switzerland) for 45 cycles (95°C for 30 s and 60°C for 5 s) after an initial 30-s denaturation at 95°C. Glyceraldehyde-3-phosphate dehydrogenase (GAPDH) was used as an internal control. The RNA levels of tumor samples and paired adjacent samples were calculated using the 2^−ΔCt^ method. All primers sequences of the hub genes and GAPDH were listed in **Supplementary Table 1**.

#### Multiplex immunohistochemistry

The expression intensity and spatial distribution of Vimentin and Nox4 in HC skin tissues and SSc lesion tissues were labeled using multiplex immunohistochemistry (mIHC). The tissue slides were melted, dehydrated, deparaffinized, and rehydrated. Then, the slides were in heat-induced antigen retrieval and were blocked. The slides were incubated with the primary antibody and secondary antibody, and tyramine signal amplification was performed. The antibodies used in this study were anti-vimentin (1:5,000, 10366-1-AP, Proteintech, USA) and anti-*Nox4* (1:100, 14347-1-AP, Proteintech, USA).

## Results

### Transcriptome patterns of fibroblasts with and without hypoxia

**Figure 2:**
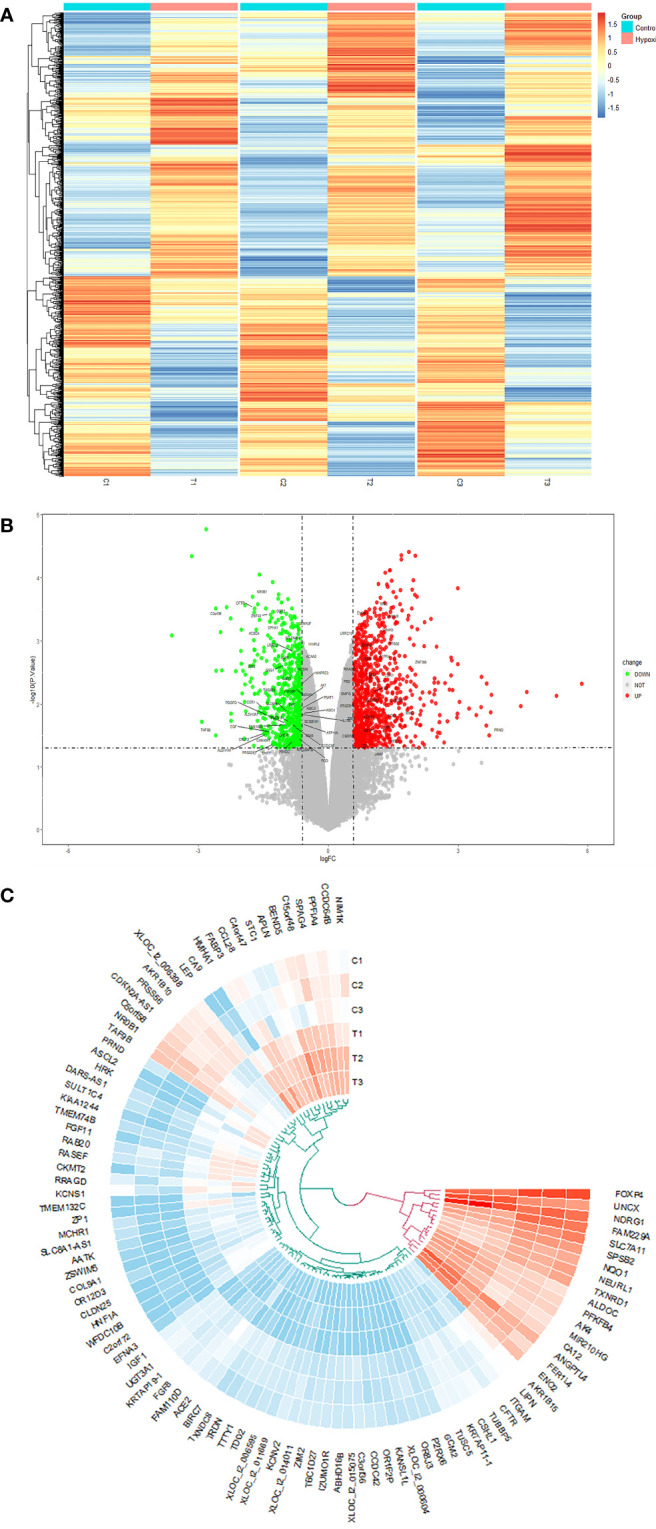
**(A)** The volcano map analysis of differential expression genes between normal fibroblasts treated with hypoxia or not. **(B)** The heat map; the red and blue points, respectively, represented upregulated and downregulated genes screened on the basis of fold change > 1.5 and a P-value < 0.05. Genes without significant difference were shown as the gray points. **(C)** Circos diagram used to show the top 50 differential genes in hyper- and hypo-expression, respectively.

There were 1,520 DEGs after hypoxia: 880 were upregulated and 640 were downregulated. The DEGs are shown in volcano map (**Figure 2A**) and cluster heat map (**Figure 2B**). In addition, we used Circos maps to show the top 50 differences in expression positions at the two peaks of the volcano map (**Figure 2C**).

In addition, we found that hypoxia can promote the proliferation of fibroblasts but has no effect on apoptosis **(** **Supplementary Figure 1** **)** and increased the expression of collagen I (COL1), COL3, TGF-β, and hypoxia inducible factor-1 alpha (HIF1-α) (**Supplementary Figure 2**). We infer that hypoxia brings fibroblasts closer *to the* myofibroblast phenotype.

### Genomic expression integration

**Figure 3:**
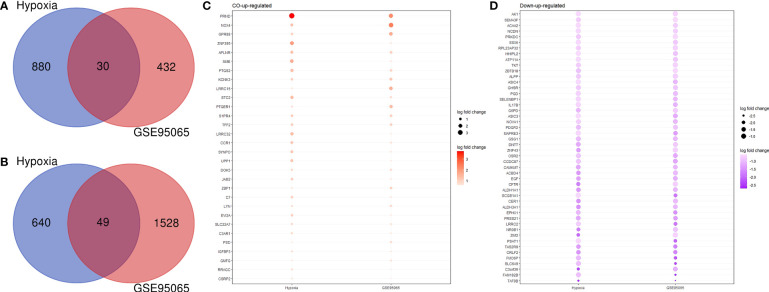
**(A)** Venn diagram shows the co-upregulated genes of hypoxia and systemic sclerosis. **(B)** Venn diagram shows the co-downregulated genes of hypoxia and systemic sclerosis. **(C)** Red and large circles indicate upregulation; **(D)** purple small circles indicate downregulation.

An analysis of the co-DEGs in the two groups of transcriptome data was used to determine the associations and interactions between hypoxia and SSc and was visualized using a Venn diagram (**Figures 3A, B**). We investigated 1,520 and 1,960 DEGs in hypoxia and SSc, respectively. Although only a small proportion of DEGs overlapped between hypoxia and SSc, the functions of these genes are important. **Figures 3C, D** present the DEGs in both hypoxia and SSc.

### Enrichment analysis of GO and KEGG pathways

We analyzed the downregulated or upregulated DEGs in the two datasets using GO and KEGG enrichment analyses.

**Figure 4:**
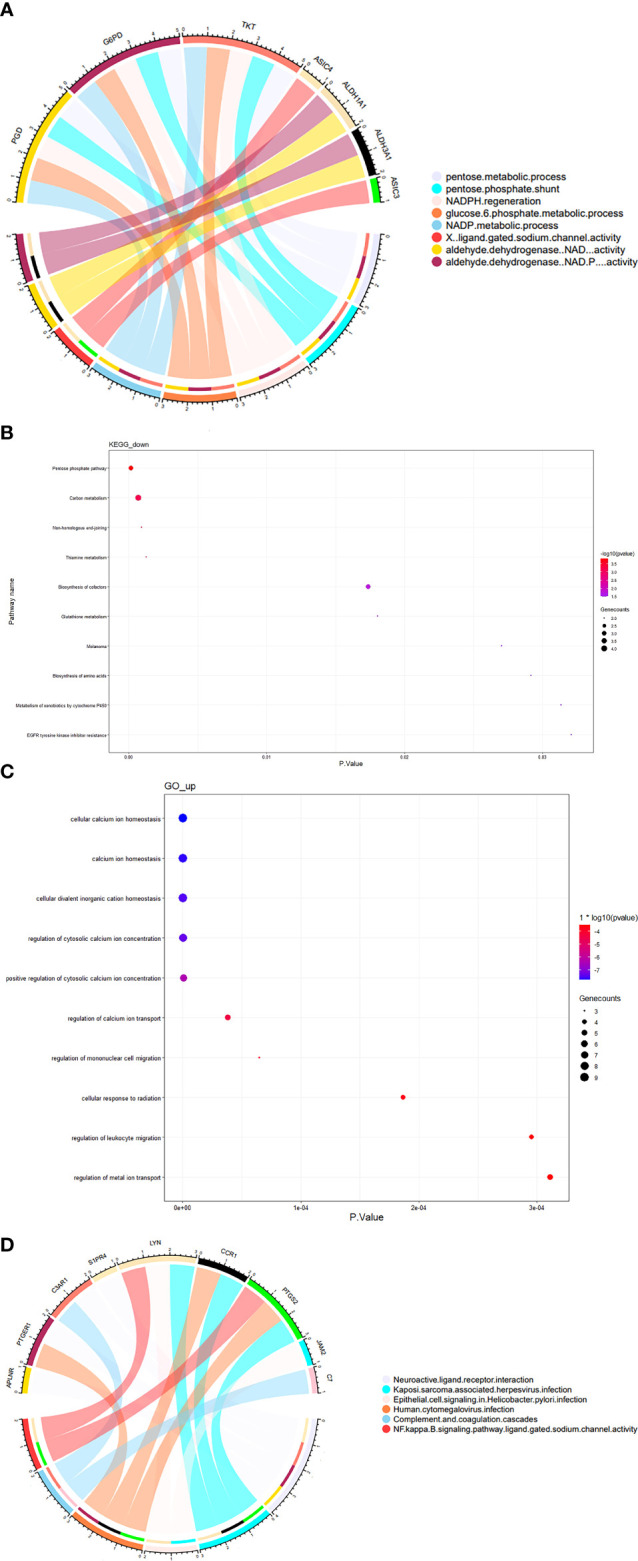
GO and KEGG enrichment analyses of co-differentially expressed genes between hypoxia-treated fibroblasts and SSc lesions. Top 10 or *p* < 0.05 terms in **(A)** chord diagram presenting the GO enrichment analysis of co-hypo-DGEs; the thickness of a band is proportional to the number of co-hypo-DGEs enriched in one GO enrichment and **(B)** KEGG pathway analysis of co-hypo-DGEs. **(C)** GO enrichment analysis of co-hyper-DGEs, and **(D)** chord diagram presenting the KEGG pathway analysis of co-hyper-DGEs. The thickness of a band is proportional to the number of co-hyper-DGEs enriched in one KEGG pathway.

GO and KEGG enrichment analyses showed that the downregulated genes in the two datasets were mainly enriched in the pentose- and nicotinamide adenine dinucleotide phosphate (NADPH)-related metabolism pathways (**Figures 4A, B**), suggesting that hypoxia affects the development of SSc by reducing NADPH production, which aggravates oxidative stress.

The GO enrichment analysis showed that the upregulated genes in the two datasets were mainly enriched in calcium ion homeostasis, concentration, and transportation (**Figure 4C**). The upregulation of signaling in calcium metabolism may explain the high occurrence of calcinosis cutis in SSc. KEGG pathway analysis showed that the upregulated genes were involved in the neuroactive ligand–receptor interaction, Kaposi’s sarcoma–associated herpesvirus infections, epithelial cell signaling in *Helicobacter pylori* infection, and human cytomegalovirus infection pathway (**Figure 4D**). These results suggest that hypoxia may be related to the increased incidence of nervous system involvement, and the aforementioned environmental factors increase the risk of development of SSc. The peripheral nervous system is frequently involved in SSc, which most commonly presents with sensory symptoms and gut dysmotility (15). Viral infections, including cytomegalovirus and human herpesvirus-6 (HHV-6), are an important risk factor of SSc development (16).

We visualized the top 10 pathways identified from GO and KEGG analyses.

### PPI network and functional expression modules

**Figure 5:**
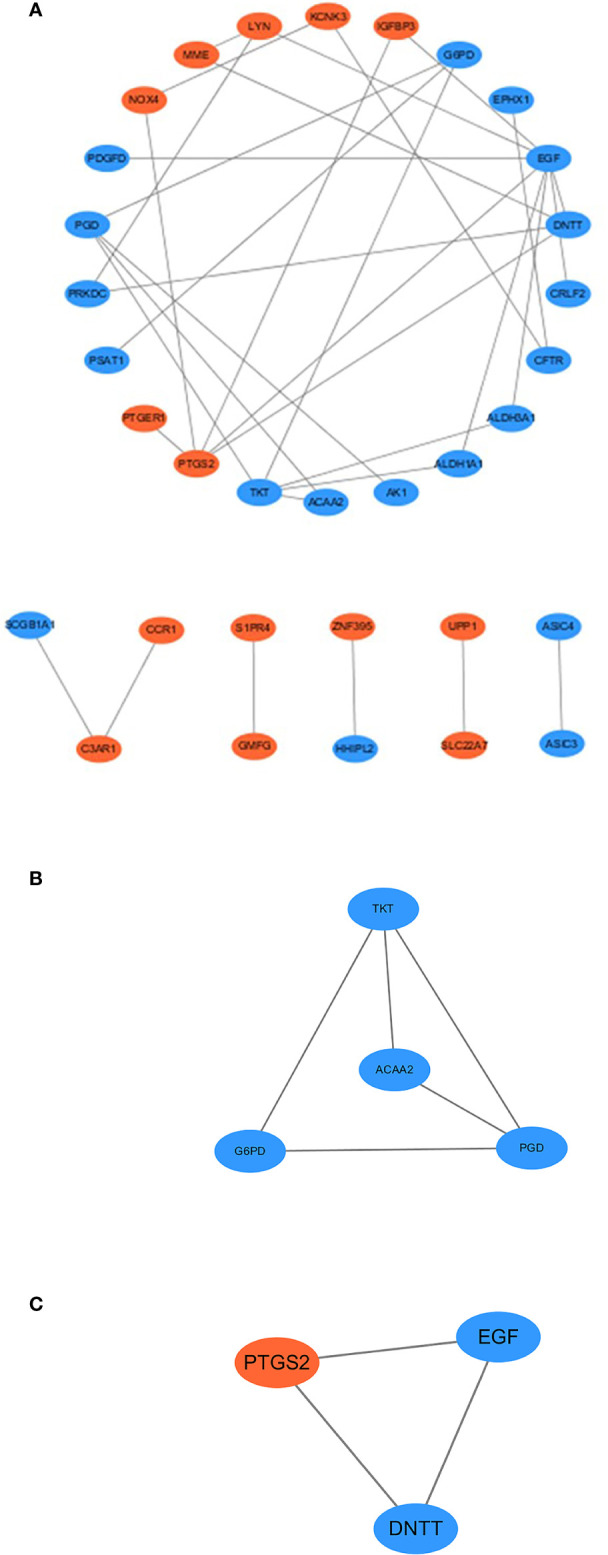
**(A)** Protein–protein interaction network of co-DEGs, with disconnected nodes hid. Blue ellipses meant downregulated DEGs, and orange ellipses meant upregulated DEGs. **(B, C)** MCODE uses vertex weighting to select the module 1 and module 2 from co-DEGs.

STRING was used to analyze the PPI network (33 nodes and 34 edges). The PPI network was visualized using Cytoscape 3.6.0. Orange and blue colors represent co-upregulation and co-downregulation, respectively (**Figure 5A**). cytoHubba, an app in the Cytoscape software, was used to select the hub genes from the PPI network. Nine of the top 10 hub genes were selected by five ranked methods in cytoHubba as being overlapped three times or more (**Table 2**). These genes were *ALDH1A1*, *EGF*, *NOX4*, *LYN*, *DNTT*, *PTGS2*, *TKT*, *ACAA2*, and *ALDH3A1*. Among them, *NOX4*, *PTGS2*, and *LYN* were co-upregulated, whereas the other genes were co-downregulated. Finally, on the basis of the PPI network, MCODE, another app in Cytoscape software, identified two important expression function modules. The enrichment analysis of functional expression modules showed that the DEGs of module 1 (**Figure 5C**) played key roles in pentose- and NADPH-related metabolism, whereas the DEGs of module 2 (**Figure 5B**) affected the process of purinergic signaling and transportation of calcium.

**Table 2:** The hub genes for DEGS ranked in cytoHubba.

Category	The hub genes for DEGS ranked in cytoHubba				
	MCC	MNC	Degree	EPC	Radiality
**Gene symbol top 5**	ALDH1A1	EGF	ALDH1A1	ALDH1A1	ALDH1A1
	EGF	NOX4	EGF	EGF	EGF
	NOX4	G6PD	NOX4	LYN	NOX4
	LYN	DNTT	LYN	ALDH3A1	LYN
	G6PD	PTGER1	G6PD	DNTT	CRLF2
	DNTT	ACAA2	DNTT	PRKDC	ALDH3A1
	ACAA2	PTGS2	ACAA2	PTGS2	DNTT
	PTGS2	PGD	PTGS2	PGD	PTGS2
	PGD	TKT	PGD	TKT	TKT
	TKT	IGFBP3	TKT	IGFBP3	IGFBP3

### Validation of the expression levels of hub genes

**Figure 6:**
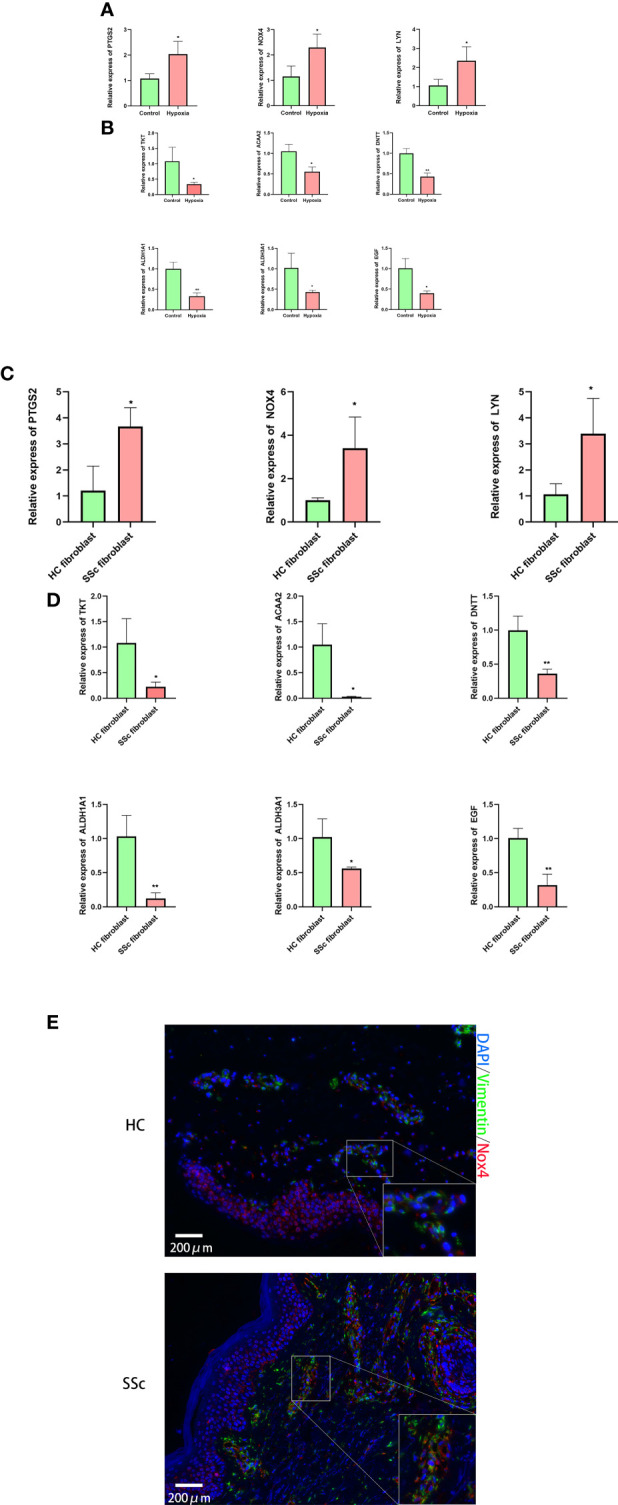
Validation of the expression levels of hub genes between in hypoxia-treated and non–hypoxia-treated fibroblasts, and between four healthy control fibroblasts and SSc fibroblasts. **(A)** Expression levels of NOX4, PTGS2, and LYN showed an upward trend in hypoxia-treated fibroblasts; **(B)** the expression levels of ALDH1A1, EGF, DNTT, TKT, ACAA2, and ALDH3A1 showed a downward trend in hypoxia-treated fibroblasts. n = 4 means four separate controls’ fibroblast treated or not in each group. **(C)** Expression levels of NOX4, PTGS2, and LYN showed an upward trend in SSc; **(D)** the expression levels of ALDH1A1, EGF, DNTT, TKT, ACAA2, and ALDH3A1 showed a downward trend in SSc. *p < 0.05, **p < 0.01. n = 4 means four separate controls’ fibroblast or patients with SSc in each group. **(E)** The merged images for of mIHC for vimentin and Nox4 in HC and SSc. n = 3 means three separate controls’ tissue sections or patients with SSc in each group.

We validated the expression levels of hub genes. By using RT-qPCR assay, we detected their expression levels between hypoxia-treated and non–hypoxia-treated fibroblasts, and between healthy control fibroblasts and SSc fibroblasts. the expression levels of NOX4, PTGS2, and LYN showed an upward trend (**Figure 6A**) in hypoxia-treated fibroblasts and SSc, and the expression level of aldehyde dehydrogenase 1A1 (ALDH1A1), epidermal growth factor (EGF), DNA nucleotidylexotransferase (DNTT), transketolase (TKT), ACAA2, and ALDH3A1showed a downward trend in hypoxia-treated fibroblasts and SSc (**Figures 6B**). By using mIHC, the expression levels of NOX4 showed an upward trend in SSc lesions (**Figure 6**).

## Discussion

SSc is a complex autoimmune disease that involves almost all organ systems. It has multiple but unclear pathogenic mechanisms, including genetic and environmental factors (15). An imbalance between the oxidant and anti-oxidant states has been suggested to initiate SSc (16, 17). Hypoxia is involved in SSc pathogenesis by inducing oxidative stress (4). However, most previous studies of the role of hypoxia in SSc have focused on fibrosis. We performed the present study to explore the role of hypoxia in SSc pathogenesis.

Previous studies have reported that hypoxia is critical for excessive extracellular matrix production and angiogenesis (18). However, we found that the upregulated DEGs in hypoxia and SSc were involved in calcium ion homeostasis, concentration, and transportation. Calcinosis cutis is a significant clinical problem that affects almost 20%–40% of patients with SSc (19). Our results showed altered expression levels of key genes involved in calcium regulatory processes, such as *C3AR1*, *CCR1*, and *APLNR*. Therefore, we hypothesized that hypoxia may directly impact calcium ion metabolism, which may lead to calcinosis cutis. This hypothesis should be tested in future studies.

In contrast to the GO analysis, the KEGG pathway analysis showed that the upregulated DEGs were significantly enriched in various pathways, including the neuroactive ligand–receptor interaction pathway and several viral and bacterial infection–related pathways. SSc frequently affects the peripheral and autonomic nervous systems (20, 21), which may manifest as dysautonomia (e.g., esophageal dysmotility and diarrhea). Dysautonomic symptoms are often related to depressive symptoms in patients with SSc (22). Our results showed that hypoxia may underlie the nervous system involvement in SSc. Furthermore, our study contributes to understanding the mechanisms underlying the environmental risk factors of SSc. Viral and bacterial infections are environmental risk factors of SSc (15). In particular, the human cytomegalovirus infection–related pathways, Kaposi’s sarcoma–associated herpesvirus infection, and epithelial cell signaling in *H. pylori* infection were involved. Cytomegalovirus infection is considered to trigger SSc. Recently, Soffritti et al. reported that cytomegalovirus infection caused altered miRNA expression in fibroblasts of genes that are differentially expressed in SSc and potentially associated with fibrosis and apoptosis (23, 24). Only a single case report published in 2017 described a patient with SSc and Kaposi’s sarcoma (25). Nevertheless, HHV-8 infection, which is associated with the development of Kaposi’s sarcoma, is common among patients with SSc, as shown by the high prevalence of positive HHV-8 Immunoglobulin G (IgG) antibody among patients with SSc (26). Similar findings have also been reported for other fibrotic diseases (27, 28). It is controversial whether *H. pylori* triggers SSc, but patients with SSc frequently have antecedent *H. pylori* infection, and *H. pylori*–negative patients with SSc have less active disease than *H. pylori*–positive patients with SSc (29, 30). Our study results suggest that hypoxia may aggravate the pathway dysfunction caused by the aforementioned factors and increase susceptibility to these environmental risk factors.

In co-downregulated DEGs, pentose- and NADPH-related metabolic pathways were significantly enriched. The pentose phosphate pathway (PPP), similar to glycolysis and tricarboxylic acid cycle, is an important part of cellular metabolism. The most important functions of PPP are converting glucose 6-phosphate into carbon dioxide ribulose 5-phosphate and NADPH production (31). NADPH is an antioxidant that can be considered as a redox equivalent in oxidative stress and reduces the production of reactive oxygen species (32). We propose that the production of reactive oxygen species (9), a notorious oxygen-derived molecule involved in SSc pathogenesis (33), is increased by decreased PPP and NADPH production. Our results provide a new sight into how hypoxia influences the SSc pathogenesis.

The DEGs in module 1 were highly enriched in the processes of pentose- and NADPH-related metabolism, whereas the DEGs in module 2 were involved in the process of purinergic signaling and transportation of calcium. The functions of both modules were relatively concentrated, similar to the GO and KEGG analyses, suggesting that these biological processes may be important for the interaction between hypoxia and SSc.

Furthermore, the PPI network showed that *ALDH1A1*, *EGF*, *NOX4*, *LYN*, *DNTT*, *PTGS2*, *TKT*, *ACAA2*, and *ALDH3A1* were the nine central genes identified by the top 10 hub nodes ranked through five ways due to gene overlapping more than three times. Among these hub genes, *NOX4*, *PTGS2*, and *LYN* were upregulated. These genes have previously been reported to play a critical role in SSc pathogenesis (34, 35). Interestingly, *PTGS2*, known as the cyclooxygenase-2 (COX-2), is the rate-limiting enzyme in the process of conversion of arachidonic acid to prostanoids (PGE2, PGD2, PGF2a, PGI2, and TAX2) (36). Multiple previous studies have shown that the activation of the COX2/PGE2 axis is an essential step in organ fibrosis, including liver (37), cardiac (38), renal (39), and skin fibrosis in SSc (40). However, in pulmonary fibrosis, COX2 has a low expression level. The exact reason for these differences is not clear. COX2 is also upregulated in other fibrotic diseases (41–43).

NADPH oxidase 4 (NOX4), a member of the NOX family, leads to Reactive oxygen species (ROS) production. The activation of NOX4 depends on its expression (44). ROS is the by-product of normal metabolism. Unfortunately, ROS overproduction may cause extensive structural damage of the cell components, including proteins, lipids, nucleic acids, and mitochondria, which leads to inflammation and fibrosis (45). ROS plays an important role in fibrosis, including liver (44, 46) and renal (47) fibrosis, as well as in SSc (48). NOX4 has been extensively studied in previous work.

LYN, a conventional member of the Src family protein kinases (SFKs), not only affects BCR signaling but also activates myofibroblasts to promote fibrosis (49, 50). In SSc, the high expression of LYN in monocytes promotes their differentiation into fibrocytes (51). In addition, KF-1607, an Src kinase inhibitor, is a potential treatment of fibrosis (52). In SSc, the upregulation of *NOX4*, *PTGS2*, and *LYN* may contribute to the development of SSc. In hypoxia, *NOX4*, *PTGS2*, and *LYN* are upregulated, which may exacerbate fibrosis in SSc.

*TKT* gene encodes an enzyme that forms a bridge between glycolysis and PPP and accelerates lipolysis at low expression levels (53). ACAA2, an enzyme of the thiolase family, plays a vital role in fatty acid metabolism and promotes the differentiation of precursor adipocytes into adipocytes (54). Both genes are related to adipocytes and have downregulated expression. The histopathology of SSc involves loss of subcutaneous adipose layers and replacement with fibrous tissue, which may lead to disease progression (55, 56). The downregulation of two genes in hypoxia may accelerate the loss of adipose tissue (57). Adipocytes are active cells filled with adipokines, including adiponectin, resistin, and visfatin, which are lost in SSc fibrosis (58, 59). The loss of adipocytes leads to loss of antifibrotic products, which explains why lipotransfer has shown excellent results in the treatment of SSc. In SSc, lipotransfer fills the atrophic areas and compensates for the lack of adipokines (60, 61). Future studies should explore whether adipocytes, in addition to vascular endothelial cells, immunocytes, and fibroblasts, also play a critical role in SSc pathogenesis. However, our results provide preliminary evidence to support this presumption.

ALDH1A1 is a member of the aldehyde dehydrogenase family. *ALDH1A1* has a low expression level in dermal dendritic cells in patients with SSc. *ALDH1A1*-positive dendritic cells resist fibrosis by introducing retinoic acid–mediated regulatory T cells. ALDH1A1 is indispensable for the conversion of vitamin A to retinoic acid (62, 63). Furthermore, the number of *ALDH1A1*-positive dendritic cells is negatively correlated with fibrosis severity in patients with SSc (64).

EGF stimulates cell migration, proliferation, and differentiation (65). As early as 1983, EGF was reported to reduce the production of collagen stimulated by TGF-β (66). Moreover, the level of EGF is decreased in the saliva, serum, and urine of patients with SSc (67). In addition, EGF receptor level is decreased in patients with SSc, and abnormal EGF pathways may be a treatment target for SSc (68).

DNTT, also known as TDT, is an important polymerase enzyme for V(D)J recombination due to its unique function of randomly incorporating nucleotides into the V, D, and J regions of heavy chains of immunoglobulins (69). In addition, the V(D)J recombination can trap naïve B cells in immaturity (70). B-cell anomalies, including increased number of naïve B cells and decreased V(D)J rearrangement frequencies, were observed in patients with SSc (71). The reduced expression of DNTT alters V(D)J rearrangement frequencies to affect B cells to participate in SSc pathogenesis.

ALDH3A1, a member of the superfamily of NAD(P)+-dependent enzymes, can oxidize diverse aldehydes into carboxylic acids (72). ALDH3A1 shows depletion in kidney fibroblasts after TGF-β treatment (73). However, other aspects of the relationship between ALDH3A1 and fibrosis are not clear. ALDH3A1 can be upregulated by nuclear factor erythroid 2-related factor 2 (Nrf2) (74). In SSc, the lack of Nrf2, a key fighter against oxidative stress, aggravates the fibrosis (75). We speculate that ALDH3A1 is vital for Nrf2-antioxidant response signaling to resist fibrosis. Therefore, ALDH3A1 can be a potent scavenger for fibrosis. The downregulation of *ALDH1A1*, *EGF*, *DNTT*, *TKT*, *ACAA2*, and *ALDH3A1* has been shown to contribute to either SSc pathogenesis or loss of adipose tissue. Similarly, in hypoxia, *ALDH1A1*, *EGF*, *DNTT*, *TKT*, *ACAA2*, and *ALDH3A1* show reduced expression levels, suggesting that hypoxia is involved in the pathogenesis of SSc.

Our study had some limitations, such as the sample size being slightly small and the transcriptome data of fibroblasts being compared with that of full-thickness skin. Although fibroblasts are not the most dominant cell type in SSc biopsies, different from keratinocytes and adipocytes, they play a critical role in the process of fibrosis through collagen synthesis, its functional and characteristic changes are the most significant and decisive in SSc, and previous studies have also shown that fibroblasts are the prominently differentially expressed cells between SSc tissues and normal tissues (76, 77). Our research had some shortcomings but largely illustrates the topic. However, the study also had several strengths. By using bioinformatics databases and tools, we found that the co-DEGs for SSc and hypoxia had different functions and involved different signaling pathways, including pentose- and NADPH-related metabolism pathways, calcium metabolism pathways, neuroactive ligand–receptor interaction pathway, and viral and bacterial infection–related pathways. The hub genes included *ALDH1A1*, *EGF*, *NOX4*, *LYN*, *DNTT*, *PTGS2*, *TKT*, *ACAA2*, and *ALDH3A1*. This study provides insights into the mechanisms underlying the effects of hypoxia on SSc pathogenesis. However, additional *in vitro* and *in vivo* functional experiments are required to confirm the genes, pathways, and hypoxia-related mechanisms involved in SSc pathogenesis.

## Data availability statement

The data presented in the study are deposited in the GEO repository, accession number GSE194296.

## Ethics statement

This study was reviewed and approved by the institutional review board of the Second Xiangya Hospital of Central South University. The patients/participants provided their written informed consent to participate in this study.

## Author contributions

XH: data curation, writing—original draft, formal analysis, and funding acquisition. YS, ZZ, BT, and JY: formal analysis, resources, and methodology. PZ, XX, and JL: software, data curation, and resources. YX and YL: investigation, project administration, and funding acquisition. RX: funding acquisition and writing—review and editing. All authors contributed to the article and approved the submitted version.

## Funding

This work was supported by the National Natural Science Foundation of China (Nos. 82073449, 82001738, and 81773333) and the Natural Science Foundation of Hunan Province, China (No. 2021JJ40273).

## Conflict of interest

The authors declare that the research was conducted in the absence of any commercial or financial relationships that could be construed as a potential conflict of interest.

## Publisher’s note

All claims expressed in this article are solely those of the authors and do not necessarily represent those of their affiliated organizations, or those of the publisher, the editors and the reviewers. Any product that may be evaluated in this article, or claim that may be made by its manufacturer, is not guaranteed or endorsed by the publisher.
