# Supplementary figures and images for: Intimate intertwining of the pathogenesis of hypoxia and systemic sclerosis: A transcriptome integration analysis
Source: Front Immunol. 2022 Oct 31;13:929289. doi: 10.3389/fimmu.2022.929289 (PMC9660309; doi:10.3389/fimmu.2022.929289)

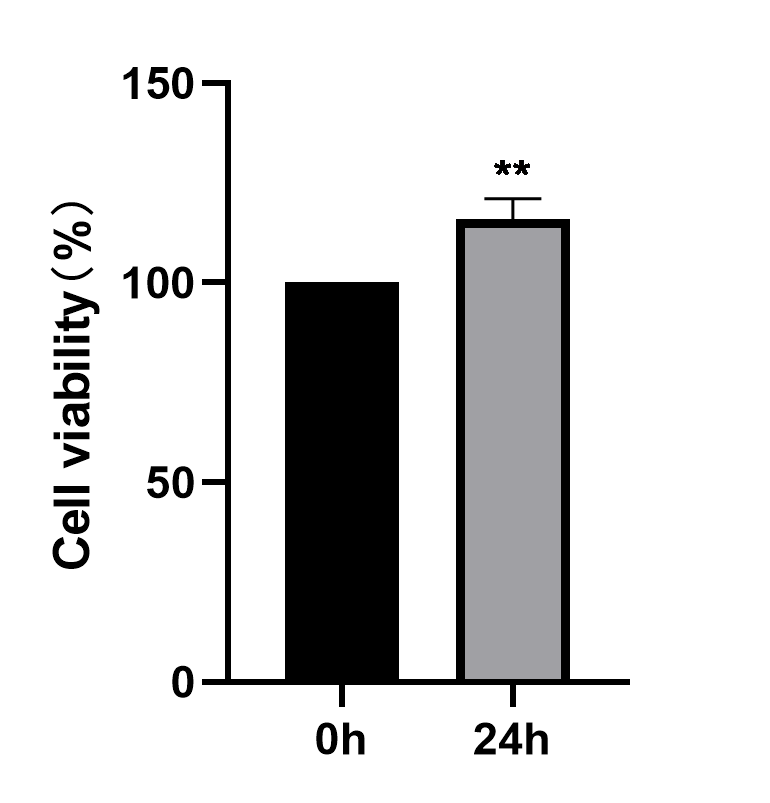

Supplement: Supplementary file 1 [file Image_1.tif]

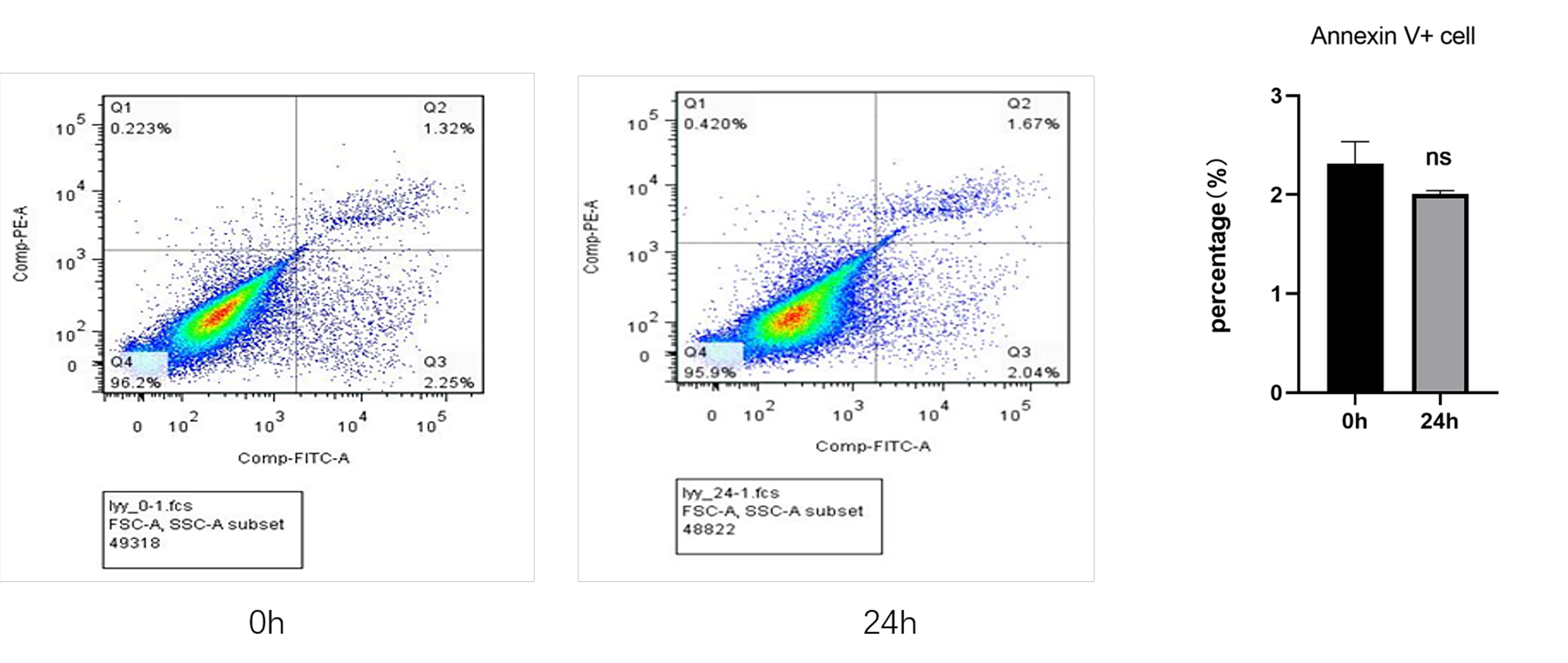

Supplement: Supplementary file 2 [file Image_2.tif]

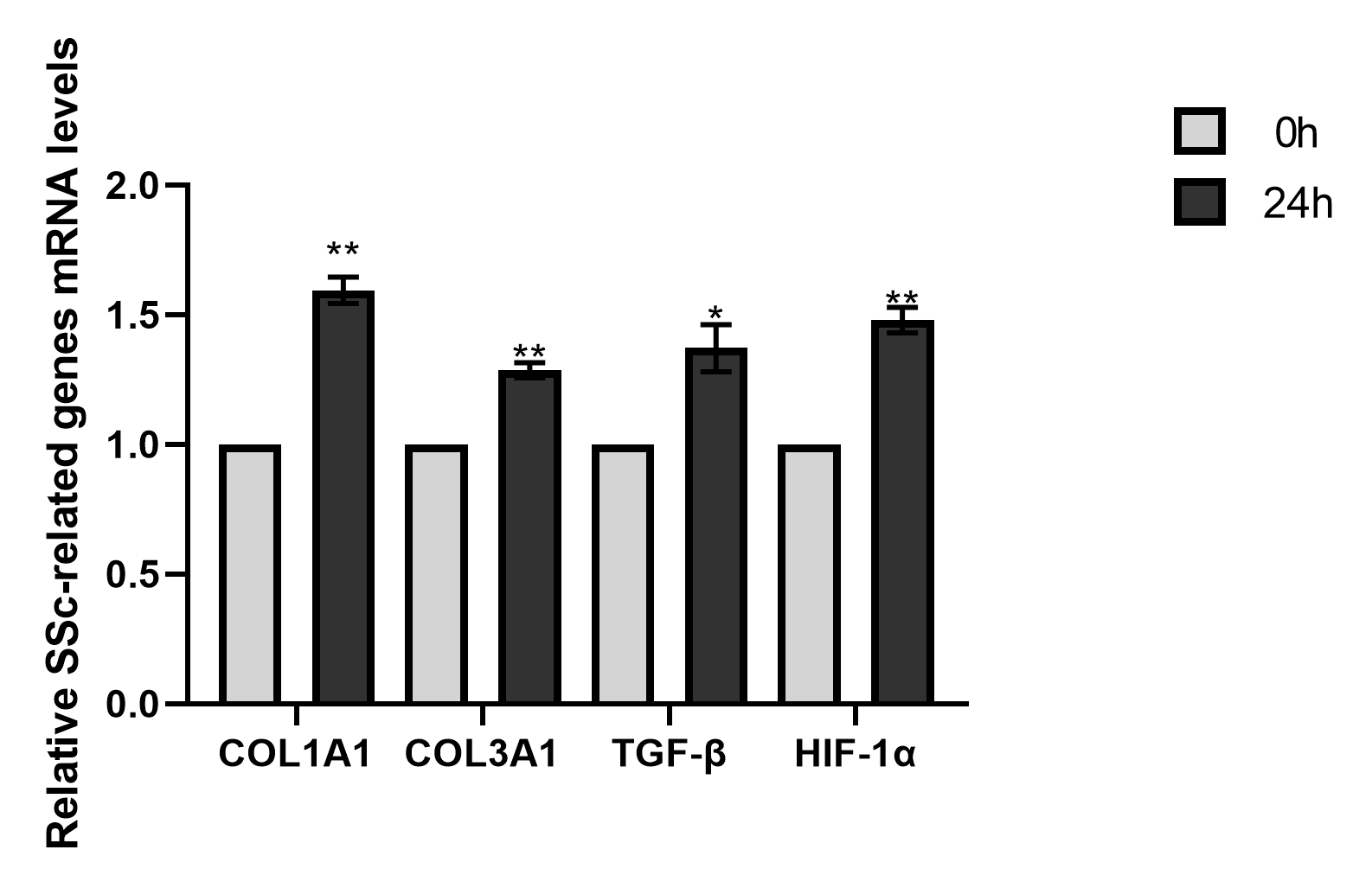

Supplement: Supplementary file 3 [file Image_3.tif]

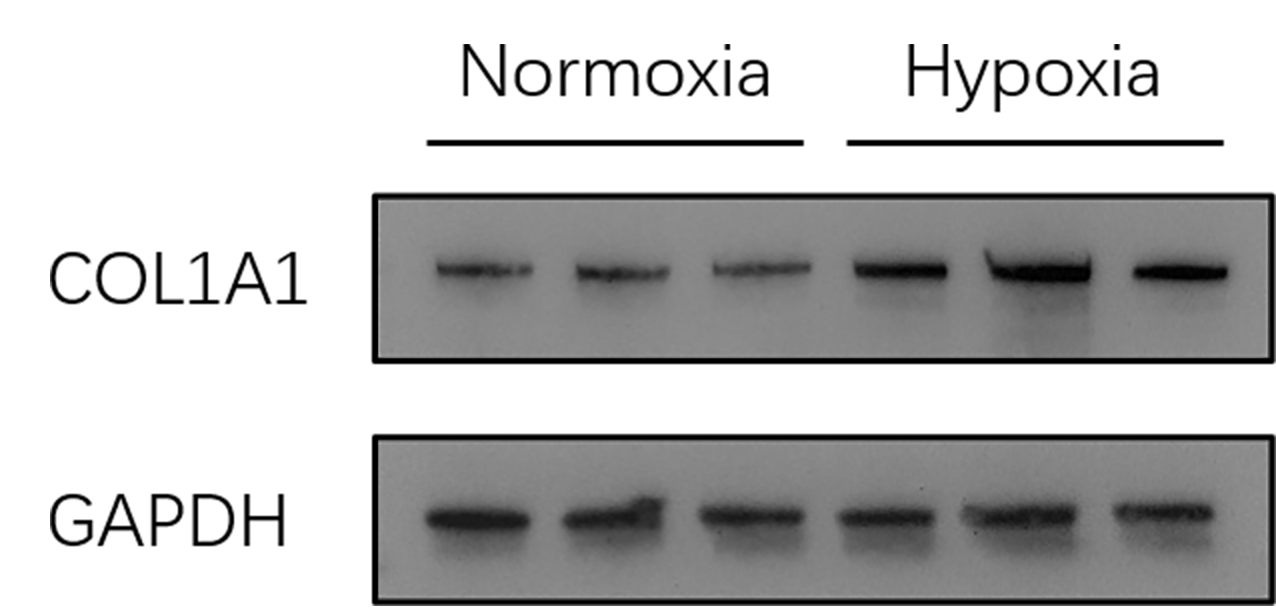

Supplement: Supplementary file 4 [file Image_4.tif]

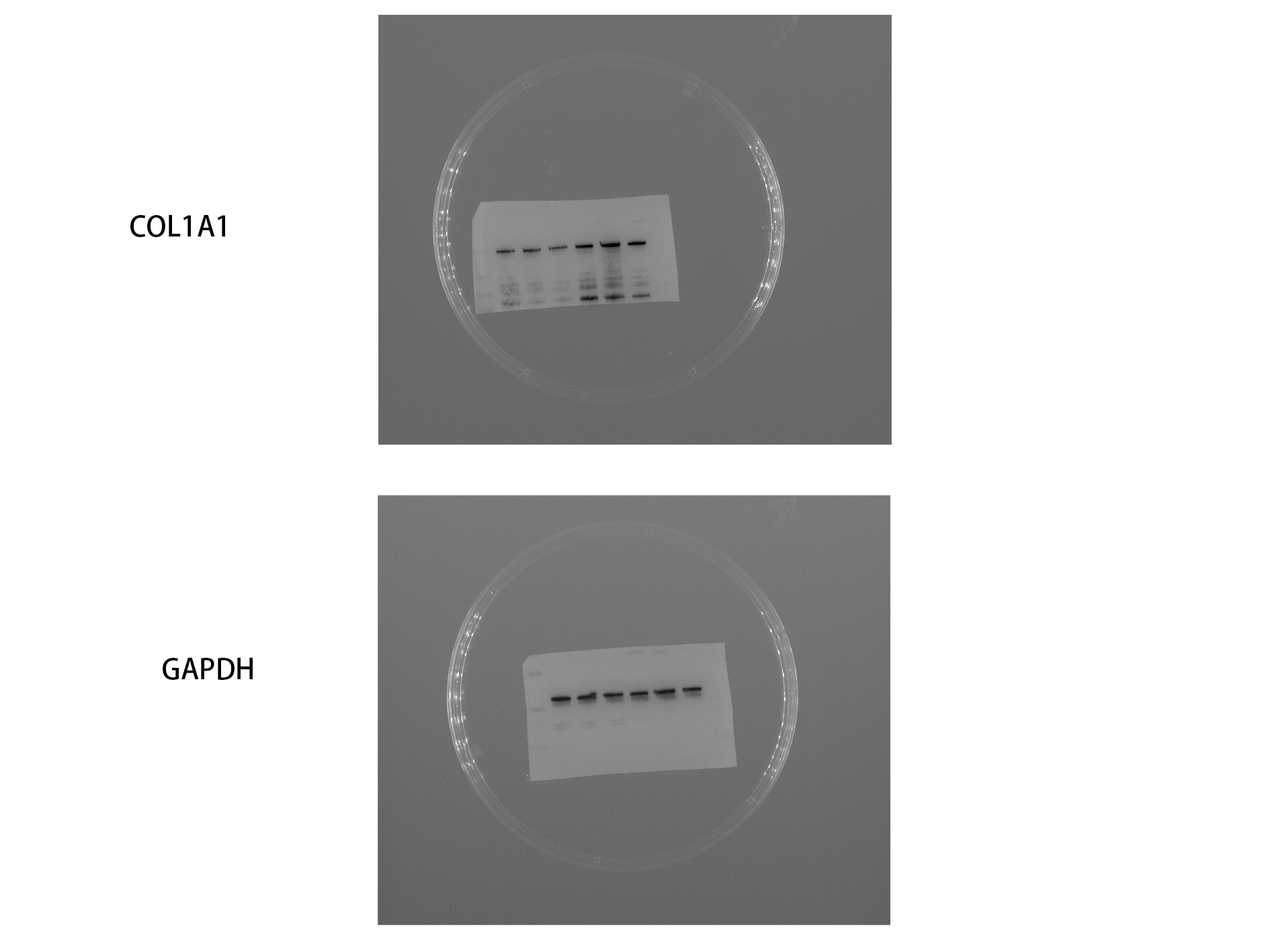

Supplement: Supplementary file 5 [file Image_5.tif]

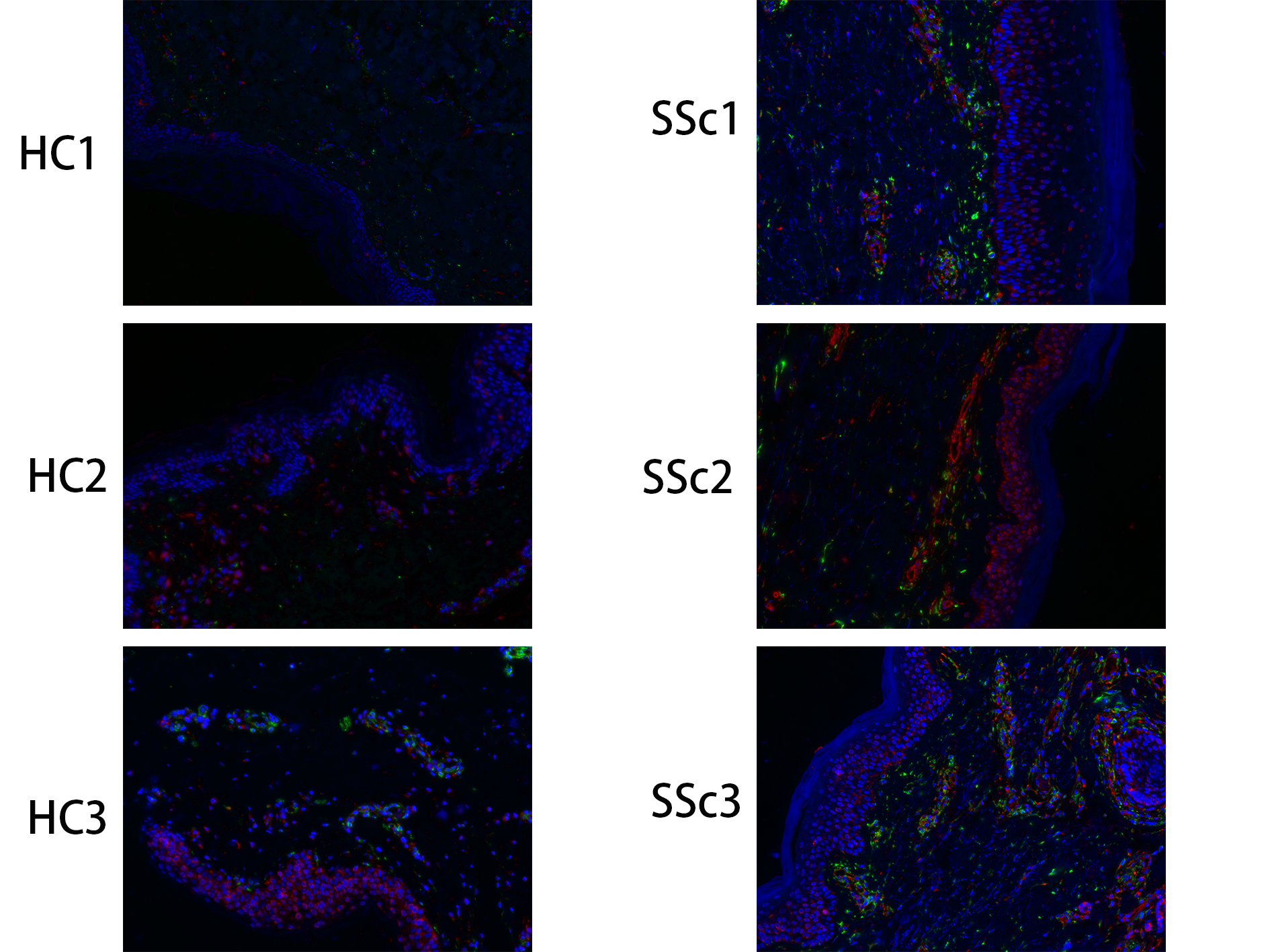

Supplement: Supplementary file 6 [file Image_6.tif]
