# Supplementary material for: Intimate intertwining of the pathogenesis of hypoxia and systemic sclerosis: A transcriptome integration analysis
Source: Front Immunol. 2022 Oct 31;13:929289. doi: 10.3389/fimmu.2022.929289 (PMC9660309; doi:10.3389/fimmu.2022.929289)
Supplement: Supplementary file 7 [file Table_1.docx]

**Supplementary Table 1**

| Gene symbol | Primer sequence |
| --- | --- |
| ALDH1A1 | F: 5′-GAGATGCGCATTGCCAAAGA-3’ |
|  | R: 5′-GCCATAGCAATTCACCCACAC-3’ |
| EGF | F: 5′-TCTACTTGTGTGGGTCCTGC-3’ |
|  | R: 5′-TCACTGAGACACCAGCATCC-3’ |
| NOX4 | F: 5′-CACCAGATGTTGGGGCTAGG-3’ |
|  | R: 5′-TGATCCTCGGAGGTAAGCCA-3’ |
| LYN | F: 5′-TAAACAGCAAAGGCCAGTTCC -3’ |
|  | R: 5′-GGGTGGATGCCATCATTAGGG -3’ |
| DNTT | F: 5′-GGGTTCCGGAGGGGTAAGA -3’ |
|  | R: 5′-TGCTAGGCAACCTGAGCTTT -3’ |
| PTGS2 | F: 5′-TTGCATTCTTTGCCCAGCAC -3’ |
|  | R: 5′-TGAAAAGGCGCAGTTTACGC -3’ |
| TKT | F: 5′-CCCGAAACAAGCTTTCACCG -3’ |
|  | R: 5′-TAGACTCGGTAGCTGGCCTT -3’ |
| ACAA2 | F: 5′-CAGGGAATGCATCGGGTGTA -3’ |
|  | R: 5′-GCCCACAATTCTTGCCAGTG -3’ |
| ALDH3A1 | F: 5′-AGGTGCTCTCTGTCCCCAG -3’ |
|  | R: 5′-GCGTTCCATTCATTCTTGTGC -3’ |
| GAPDH | F: 5′-GGAGCGAGATCCCTCCAAAAT -3’ |
|  | R: 5′-GGCTGTTGTCATACTTCTCATGG -3’ |
